# Word and Face Recognition Processing Based on Response Times and Ex-Gaussian Components

**DOI:** 10.3390/e23050580

**Published:** 2021-05-08

**Authors:** Carmen Moret-Tatay, David García-Ramos, Begoña Sáiz-Mauleón, Daniel Gamermann, Cyril Bertheaux, Céline Borg

**Affiliations:** 1 Escuela de Doctorado, Universidad Católica de Valencia San Vicente Mártir, 46002 València, Spain; 2 MEB Laboratory, Faculty of Psychology (Sede Padre Jofré), Universidad Católica de Valencia San Vicente Mártir, 46100 València, Spain; 3 Dipartimento di Neuroscienze Salute Mentale e Organi di Senso (NESMOS), Università Sapienza di Roma, 00185 Roma, Italy; 4 Study Abroad Program & Facultad de Magisterio y Ciencias de la Educación, Universidad Católica de Valencia San Vicente Mártir, 46110 València, Spain; david.garcia@ucv.es; 5 Escuela Técnica Superior de Ingeniería del Diseño, Universitat Politècnica de València, 46022 València, Spain; bsaizma@ega.upv.es; 6 Instituto de Física, Universidade Federal do Rio Grande do Sul (UFRGS), Av, Bento Gonçalves 9500, 15051, CEP 91501-970 Porto Alegre, Brazil; danielg@if.ufrgs.br; 7 Centrale Lyon ENISE, LTDS, University of Lyon, UMR 5513 CNRS, 58 Rue Jean Parot, CEDEX 02, 42023 Saint-Etienne, France; cyril.bertheaux@enise.fr; 8 Neuropsychology, Department of Neurology, University Hospital of Saint-Etienne, 42270 Saint-Etienne, France; celine.borg@chu-st-etienne.fr; 9 Psychology Department, University of Lyon, 23 Place Carnot, 69002 Lyon, France; 10 Psychology and Neurocognition Laboratory, CNRS UMR 5105, University of Savoie Mont-Blanc, 73001 Chambery, France

**Keywords:** face recognition, word recognition, celebrities, ex-Gaussian fit, reaction time

## Abstract

The face is a fundamental feature of our identity. In humans, the existence of specialized processing modules for faces is now widely accepted. However, identifying the processes involved for proper names is more problematic. The aim of the present study is to examine which of the two treatments is produced earlier and whether the social abilities are influent. We selected 100 university students divided into two groups: Spanish and USA students. They had to recognize famous faces or names by using a masked priming task. An analysis of variance about the reaction times (RT) was used to determine whether significant differences could be observed in word or face recognition and between the Spanish or USA group. Additionally, and to examine the role of outliers, the Gaussian distribution has been modified exponentially. Famous faces were recognized faster than names, and differences were observed between Spanish and North American participants, but not for unknown distracting faces. The current results suggest that response times to face processing might be faster than name recognition, which supports the idea of differences in processing nature.

## 1. Introduction

When we are introduced to someone, we try to remember attributes that allow further identification, such as names or facial features. However, names are remarkably more difficult to learn and remember than other attributes [1]. This can be explained from theories related to the entropy approach. A recognition process might reach a maximum entropy when all stimuli are equally likely, and reduce it when some stimuli are more noticeable than others [2]. In this way, changes in response times are proportional to the entropy of the signal source [3]. This is of interest for different fields, where the nature of face and name recognition, its differences, and similitudes, still remains a controversial topic.

Since the 1990s, numerous contributions in the field of neuroscience have shown that, even if the gyrus fusiform is shared for both word and face recognition, the areas of the brain involved in visual recognition vary according to the type of visual stimuli. Especially when it comes to recognizing faces, objects, or words [4,5]. The fusiform area (FFA) in the mid portion of the fusiform gyrus is involved in the processing of unique facial identity [6], while the visual form area (VWA) is another mid-fusiform regions that processes a selection for visually presented words [5]. The FFA gives a larger response in the right hemisphere whereas the VWA [7] activates the left hemisphere [8]. Such findings are also confirmed by event-related potential (ERP) and magnetoencepholography (MEG) studies [9]. In addition, studies in neuropsychology shows that the impaired face recognition of prosopagnosia is the result of a damage in bilateral or right occipito-temporal cortex [6], while the impaired reading alexia is associated with a left occipito-temporal damage [10,11]. 

The nature of word and face recognition appears to be remarkably complex. Faces are multidimensional visual stimuli bringing a lot of information organized into two categories: faces traits and face states. Face traits refers to a stable and permanent processing such as the processing of facedness (face or non-face), species (humans or animals), gender (male or female), race (Chinese or Caucasian), age (old or young), and identity (Carmen or Cyril). Faces states refer to dynamic and transient facial cues such as emotional expression [12]. Viewing a face generates automatic and fast processes: categorization of the stimulus as face, belonging to a group or not, and recognition of the face with its specific features [13,14]. The specific function of the VWA would be, for much of the literature, the visual recognition of chains of letters within the processing belonging to the expert reader [8]. Other studies explored the time course of word, object, and face recognition using event-related potentials (ERPs). Comparison analyses show periods of activation at 100 ms for faces and at around 200 ms for objects and words [15]. The recognition of human faces has been considered an innate, and quicker than word recognition, process that must be learned, and therefore processed slower. Another study has shown that signals associated with different facial identities can be discriminated as early as 70 ms after stimulus presentation [16], but this time may vary depending on whether it is a known or unknown face. It is the same case for word recognition, which may vary depending on word length, frequency, and semantic coherence of a word’s morphological family [17]. A magnetoencephalographic study of word recognition places letter recognition at approximately 200 ms and lexical recognition between 300 and 390 ms depending on their complexity [18]. 

Another question addressed in the literature is whether face identification engages specific attentional and executive mechanisms. Three points of view are expressed. The first is that face recognition is automatic and requires no attention [19]. This point of view relies on the level of faces familiarity. A second point of view referred to the existence of separate attentional resources underlying featural and perceptual mechanisms and that optimal face processing engage a specific attentional resource allocated towards configural processing [20]. In this way, other authors suggest that holistic processing could also be automatic [21,22,23]. A third point of view suggests that attention is needed to process faces in the same way as is needed for any other stimuli. Nevertheless, this point of view has a limit, a feature lacking in the suggestion of separate mechanisms for configural vs. featural processes [24]. If these results are compared with the processing of written stimuli, the literature seems to be more explicit towards attentional independence. It should be noted that the VWFA has been described as part of the language and attention circuits [25]. 

In addition, important advancements have made in the domains of familiar faces recognition. The first cognitive model that has tried to analyze the functional architecture of processes underlying the famous people is the one proposed by Bruce and Young [26]. They identified different stages of processing, from a three-dimensional structural description to a modality specific “face recognition units” allowing access to the identity of famous people. Another model [27] is particularly interested in the nature of semantic knowledge on persons. In this model, two types of stimuli, a face, or a written name, can be processed to allow the recognition and then the identification of this person. Other authors [28] showed that a patient with left temporal lesions appear unable to identify a person from their name, but can identify the same person from their face. In general populations, other factors have been described that may interact with face recognition, including place of residence or gender roles [29,30]. The underlying explanation lies in the number of stimuli we are confronted with. Therefore, people who live in bigger cities might be more exposed to a wider range of stimuli, training this process. Research in the comparison with different population is of interest for this reason.

The aim of this work and its underlying research question is therefore to examine which process is produced earlier, the treatment of name or the treatment of a face, through a masked priming paradigm. It has been suggested that effects in this technique may be pre-lexical in visual word recognition tasks. In this way, some authors have found priming effects that were equally robust for words and non-words [31,32]. Another study has found similar results on same-different tasks applied to strings of digits and symbols [33]. This could be of interest for the comparison of two related but different stimuli, such as faces and their associated names. Previous studies based on cognitive tasks have employed network analysis to illustrate the connectivity between word and name recognition processes [34]. A supplementary strategy to this approach is proposed in this work: the exponentially modified Gaussian distribution (ex-Gaussian). This distribution is the result of an exponential and a normal distribution by employing and combining different components (*µ*, *σ*, and *τ*) [35]. One should bear in mind that the dependent variable proposed in this study, the response time, is characterized by its positive skewness. For this reason, trimming techniques or transformation, are often used for normalizing positively skewed data [36]. However, an ex-Gaussian fit might be an alternative that allows us to employ all data instead of trimming it, as well as to use it without any kind of transformations [37]. In sum, it is hypothesized that earlier effects occur for face recognition in both response times and ex-Gaussian components.

## 2. Materials and Methods

The study was carried out in accordance with the Helsinki Declaration. Thus, in order to participate in the different studies, all participants gave written informed consent (approval of the committee UCV/2017-2018/31). All of them were university students, with a high understanding of Spanish, and in an age range between 18 and 30 years. However, as international student groups were included, instructions and information regarding the task under study were also included in the language of their mother tongue. They always participated on a voluntary basis.

### 2.1. Participants

The inclusion criteria were, being a university student and belonging to the groups of countries of interest (Spain and USA). All participants were evaluated in Spain. Students from USA were in their first week from an international program and were from Alaska, Colorado, Georgia, Idaho, Iowa, Kansas, Kentucky, Maryland, Massachusetts, Michigan, New Jersey, New York, North Carolina, Oklahoma, South Carolina, South Dakota, Tennessee, Virginia, and Wisconsin. The Spanish participants were from Andalucía, Aragón, Asturias, Castilla la Mancha, Cataluña, Comunidad Valenciana, Extremadura, Madrid, and Murcia. In reference to the exclusion criteria, all students reported no history or evidence of neurological or psychiatric disease or not to be a Spanish or English native speaker in the country of reference. Most of the participants were right-handed (only three University students were left-handed). Sample sizes were previously calculated with the G*Power Software [38] for mixed or simple designs of repeated measurements. Note that these types of designs require smaller sample sizes because all participants go through all conditions [39]. An average expected effect size (0.25) was selected. A total of 100 university students participated in this study, divided into two subgroups: a total of 50 Spanish students (12 men and 38 women) and 50 North American students (9 men and 41 women). 

### 2.2. Stimuli

For the purpose of employing a categorization task, a list of celebrities or international reference persons published in previous studies was selected [40,41,42]. Appendix A depicts the material characteristics adapted from previous studies. All the stimuli were presented in black and white resolution. Participants were instructed to identify international celebrities or personalities by name or face and discard unknown stimuli. In the study two simple tasks were used with 28 test stimuli and 28 distractors with repeated measures (with a final total of 224 stimuli), selected from a previous study with University students from Brazil, Spain, and the USA [34]. Google frequency searches were also employed as a measure of frequency of the stimuli, as suggested in previous literature [40,43,44].

### 2.3. Procedure

**Figure 1:**
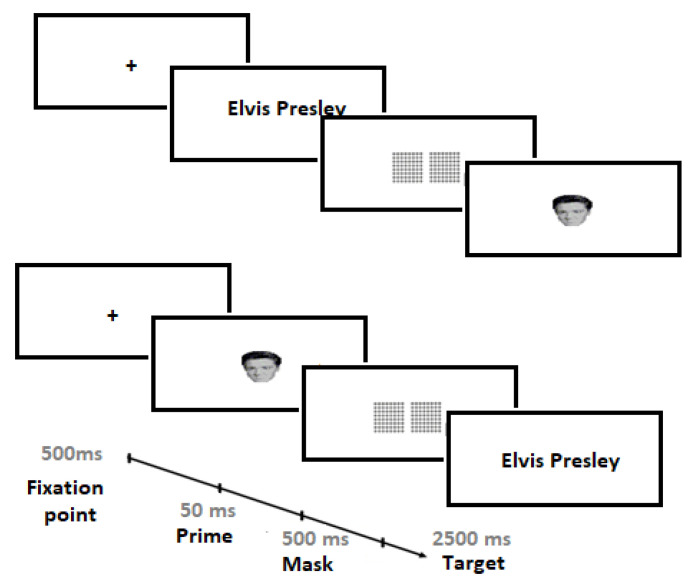
Examples of the conditions related to the masked priming task. At the top, an example of a block for the Prime (Celebrity name)–Test stimulus, and vice versa at the bottom. Blocks were counterbalanced in all groups.

A recognition task with masked priming was selected. Participants were tested in an isolated room, where the participants were assessed in groups. Two labels were included on the keyboards, the letter M a green one and the Z a red one. Participants were instructed to press the green key to indicate an identification of a test stimulus, and the red key to discard a distracting stimulus. A masked priming task was employed. Thus, after the presentation of the fixation point (+) for 50 ms, a prime stimulus (50 ms) was briefly presented and a mask (500 ms) preceded the previous stimulus. Finally, the Test stimulus was presented, with a maximum time of 500 ms. Figure 1 shows an outline of a typical trial. Each session lasted approximately 20 min.

We could describe the conditions used as follows: (i) Identity condition, where the Prime was the same stimulus as the test stimuli, (ii) related condition, where the Prime was a name related to the test stimuli both from the same celebrity, but with different natures (as a result, it can be given in two forms Face/Name or Name/Face), (iii) unrelated condition, where the Prime was a name not related to the test stimuli, and with different natures (again, it can be given in two forms Face/Name or Name/Face).

### 2.4. Design

The experiment was developed under a Target (two levels Face or name) × Country (two levels Spain or USA) × Test (two levels Face or name) × Prime (four levels across Identity, related prime to the celebrity/name or face, unrelated prime from same and different nature) design. A trimming or cut-off of the response times below 250 ms and above 1500 ms was used (always trying to exclude a number less than 5% of the responses). In addition, response times corresponding to incorrect responses were excluded from the analysis. Each experiment was preceded by practice trials (with characteristics similar to the experiment). Therefore, a classical analysis of variance (ANOVA) was performed on the response times of the correct answers and the accuracy or hit rate of the participants. The analyses were performed with the SPSS v.23 (IBM) statistical packages, and scripts created specifically in Pyhton [45] with the support of Gnuplot 4.6. The following is a description of the relevant aspects of each study.

## 3. Results

As depicted in Table 1, data were addressed in terms of average *M*, standard deviation *S* and *t*, a skewness parameter described in previous literature to depict skewness [45], as well as the ex-Gaussian components (*µ*, *σ*, and *τ*). The Shapiro–Wilk normality test was used to examine whether the trimmed variables were normally distributed, with a threshold of *p* > 0.05. The ANOVA on the response time (RT) for the test stimuli showed that faces were processed faster than names: F(1,98) = 20.57; MSE (Mean squared error) = 16026.26; *p* < 0.001; *η*^2^ = 0.17. Moreover, stimuli in the identity condition were processed faster than the stimuli in other conditions: F(3,294) = 84.46; MSE= 1522.93; *p* < 0.001; *η*^2^ = 0.46. An interaction was found for the nature of stimuli (faces versus names) X group (Spain versus USA): F(1,98) = 9.38; MSE = 150375.30; *p* < 0.01; *η*^2^ = 0.09. With regard to accuracy, statistically significant differences were found in the stimuli in the related condition: F(3,294) = 14.71; MSE = 0.03; *p* < 0.001; *η*^2^ = 0.13. This effect interacted with the nature of the stimuli (faces versus names) X group (Spain versus USA): F(3,294)= 15.64; MSE = 0.04; *p* < 0.01; *η*^2^ = 0.14. 

**Figure 2:**
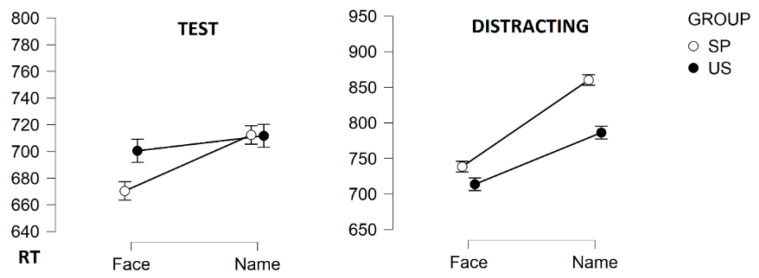
Interactions between response times in face (*SD* = 77.43) and name (*SD* = 90.97), as well as responses for face (*SD* = 88.64) and name distracting ones (*SD* = 111.95) for Spain (SP). Interactions between response times in face (*SD* = 92.26), and name (*SD* = 110.39), test stimuli, as well as responses for face (*SD* = 91.96) and name distracting ones (*SD* = 117.88) for USA (US).

On the other hand, the ANOVA on distracting TRs showed that target faces were processed faster than target names: F(1,98) = 106.74; MSE = 23153.39; *p* < 0.001; *η*^2^ = 0.52. Likewise, stimuli in identity prime conditions were processed faster than stimuli in other conditions, but with a smaller effect than responses to test stimuli: F(3,294) = 10.58; MSE = 19811.22; *p* < 0.001; *η*^2^ = 0.09. Again, an interaction was found for Target stimuli (faces versus names) X Test: F(1,98) = 17.97; MSE = 38514.52; *p* < 0.01; *η*^2^ = 0.15. Interactions found in response times, for both test and distracting stimuli, were depicted in Figure 2. Note that no interaction by Country was found. In relation to accuracy, statistically significant differences were found for the related prime condition: F(3,294) = 16.91; MSE = 0.03; *p* < 0.001; *η*^2^ = 0.14. In addition, the target names were recognized more efficiently than the target faces: F(1,98) = 7.15; MSE = 0.04; *p* < 0.01; *η*^2^ = 0.06.

**Figure 3:**
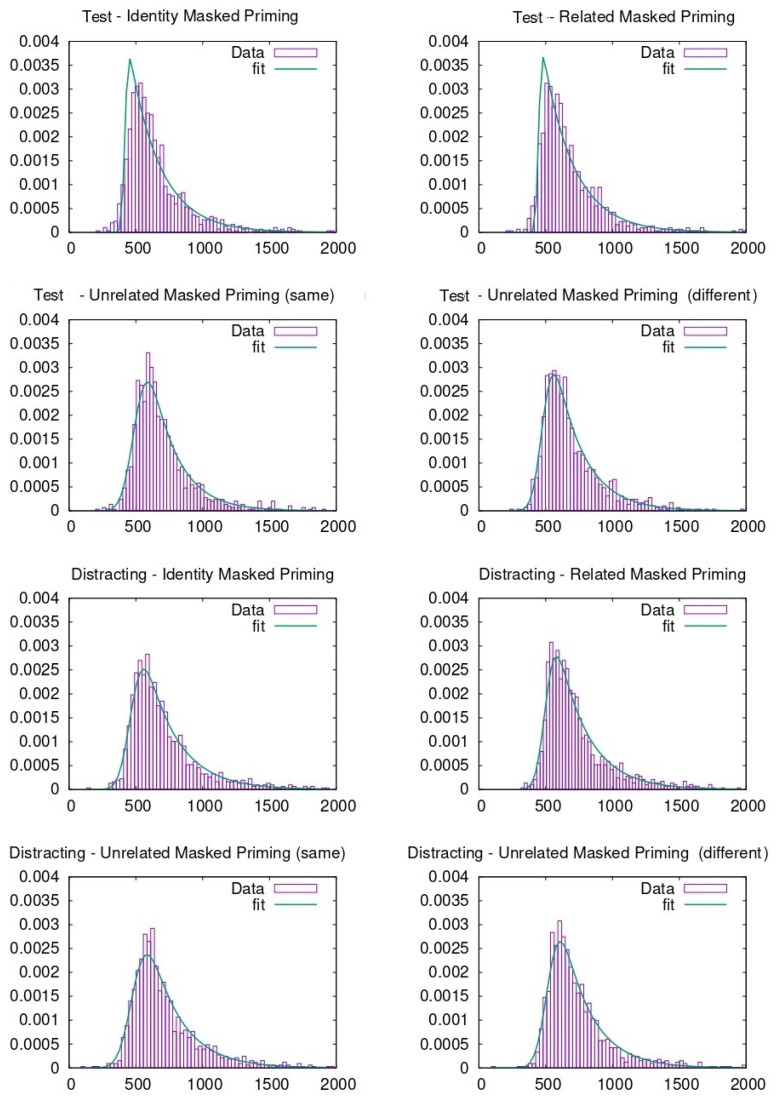
Ex-Gaussian fits carried out on face recognition data when participants were from Spain.

**Figure 4:**
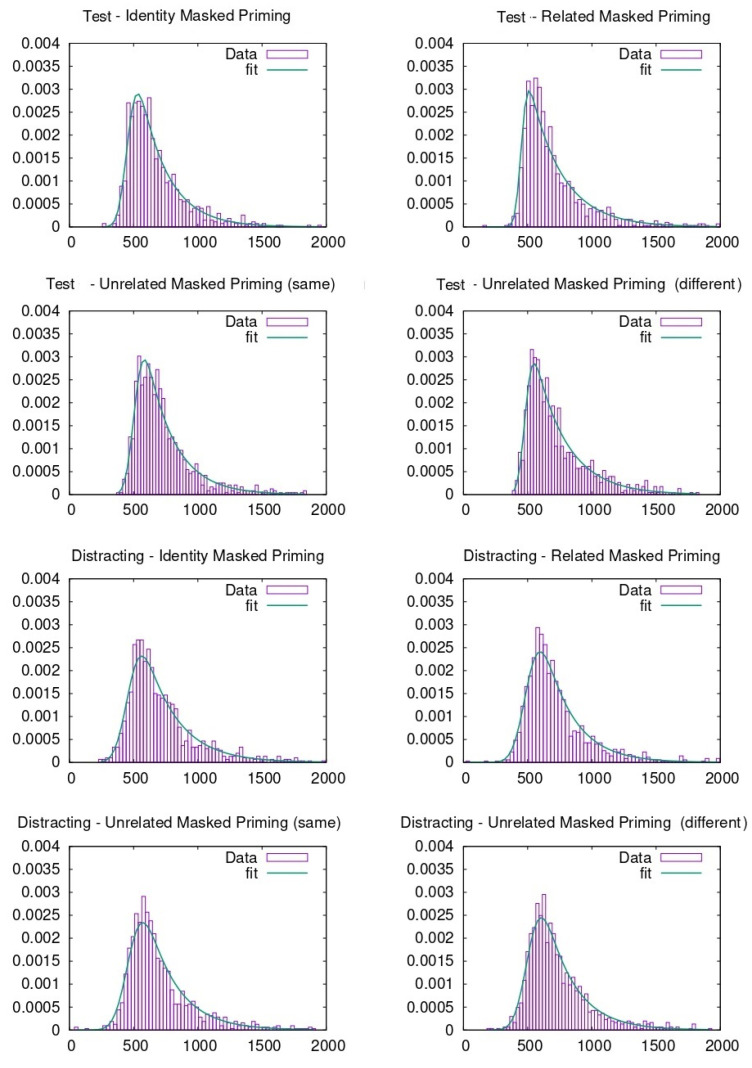
Ex-Gaussian fits carried out on face recognition data when participants were from the USA.

**Figure 5:**
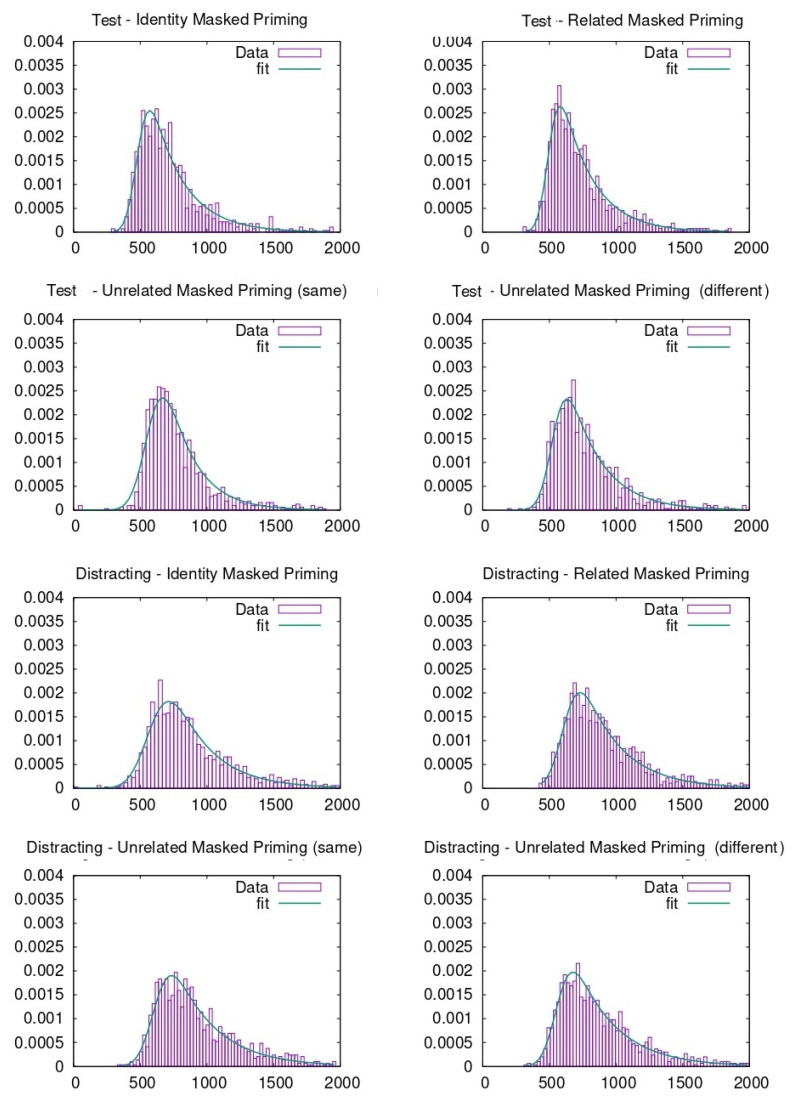
Ex-Gaussian fits carried out on name recognition data when participants were from Spain.

**Figure 6:**
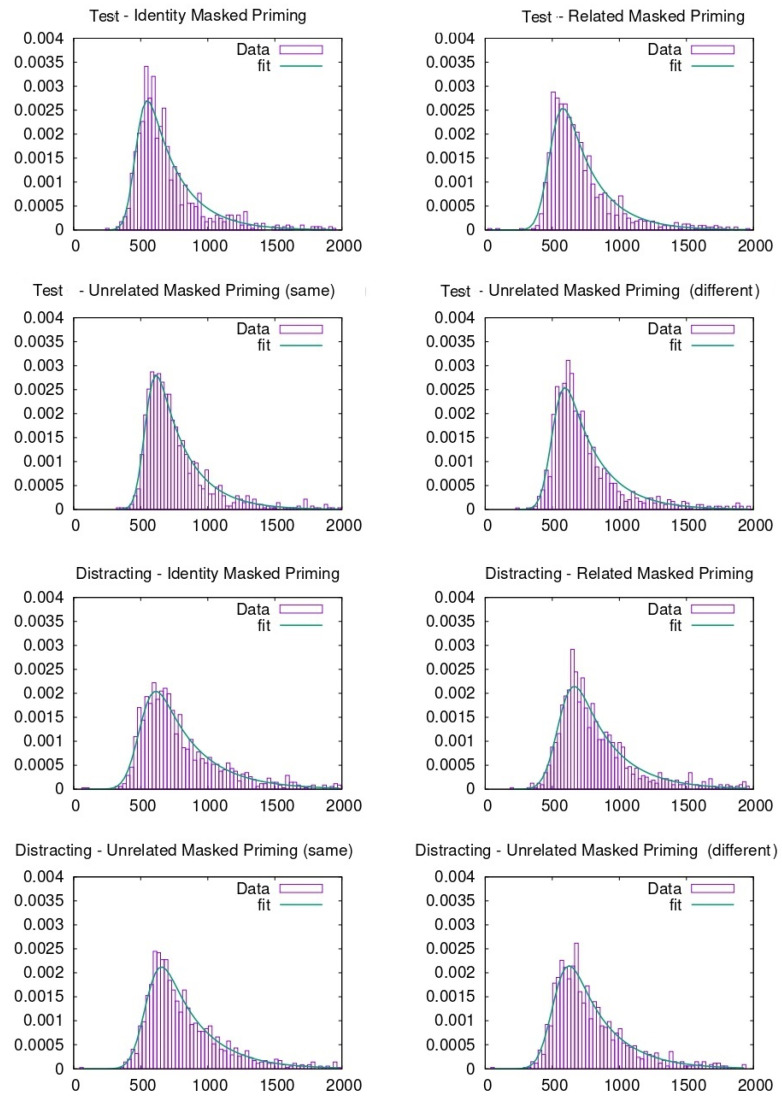
Ex-Gaussian fits carried out on name recognition data when participants were from the USA.

RTs were pooled together to carry out the ex-Gaussian fit. To do so, different methods can be adopted, as described in previous literature [46]. In our case, the p-value evaluation follows the same procedure explained in previous literature through the python package denominated ExGutils [45]. In this way, a maximum ascent algorithm in parameter space searches for the values of µ, σ, and τ that maximize the likelihood of the observed dataset. The starting point of the search are the parameter values corresponding to the dataset statistics *M*, *S*, and *t* and the search stops when the modulus of the likelihood gradient in parameter space is below *ε* = 10^−8^. Although the plots were made with histograms to visualize the data (see Figure 3, Figure 4, Figure 5 and Figure 6), the fitting procedure is independent of any parametrization and uses the raw values for all response times considered in each dataset. The *p*-values in the tables are the probability (evaluated by bootstrap from 1000 samples) that a sample of the same size as the dataset obtained from an ex-Gaussian distribution with the adjusted parameters has a KS-statistic bigger than the one obtained between the dataset and the adjusted ex-Gaussian. Therefore, the bigger this *p*-value is, the better the obtained fit was.

**Figure 7:**
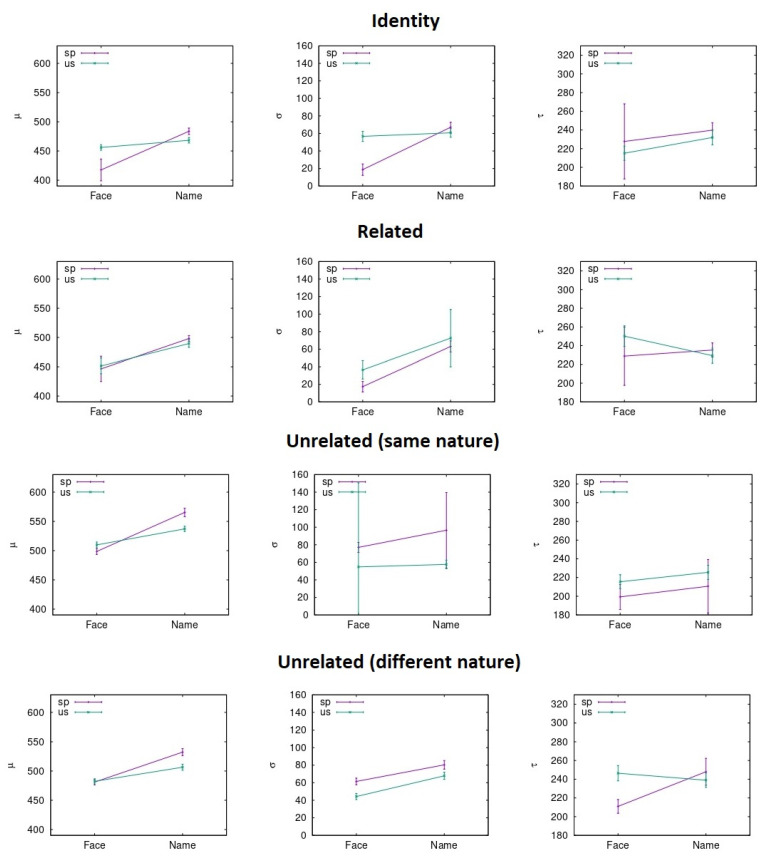
Ex-Gaussian parameters average across prime conditions and countries for the response times in the test condition.

**Figure 8:**
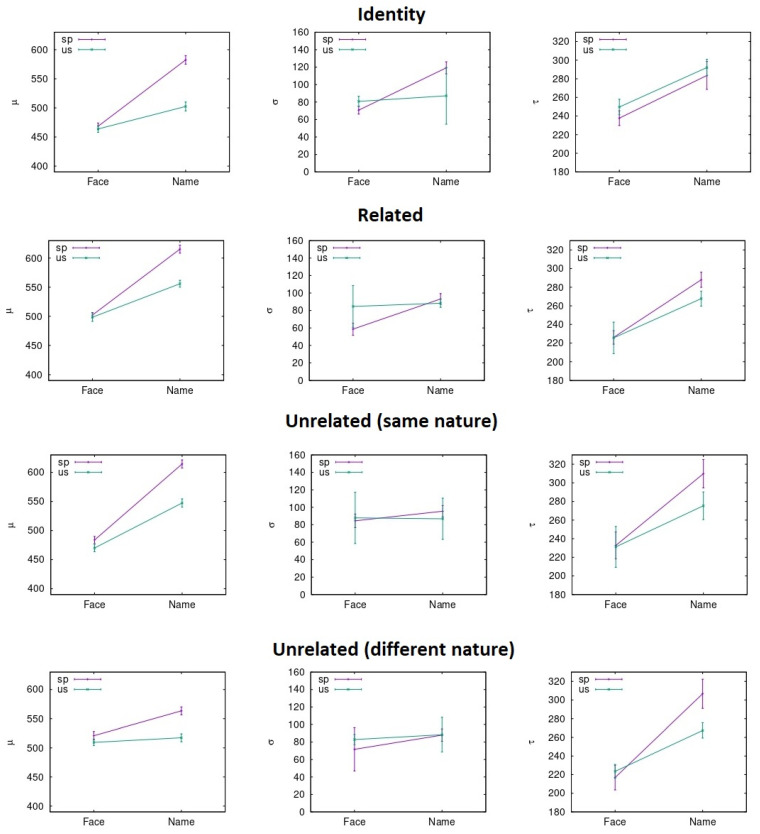
Ex-Gaussian parameters average across prime conditions and countries for the response times in the distracting condition.

In parameter space (3D space where the axes are *µ*, *σ*, and *τ*), every point that results in a likelihood half a point below the maximum is within the ∼68% region of confidence level. After sampling more than 1000 points of this surface for each dataset related to each condition under study, the uncertainties were considered as the standard deviation (dispersion) for the parameter value and for the sample points in this surface [47]. This number was multiplied by 1.96, to get a 95% confidence level interval (as in the current case for Figure 7 and Figure 8 with this interval shown in the error bars).

## 4. Conclusions and Discussion

The aim of this study was to examine differences between face and word recognition through response and time components. These two processes have been compared among each one, being considered as two sides of the same coin in the literature [48]. Let us remember that both processes share a similar area in the brain with, some specializations in the fusiform gyrus, and that both are examples of expert visual processing. On the other hand, the role of certain internal variables, such as hometown, has been studied, as a major source of variability for the recognition process [29,30].

The main results can be listed as follows: (i) Faster response times were found for responses to face than name stimuli; (ii) participants from Spain were faster in face recognition tasks, slower for name recognition ones, and vice versa for participants from the USA in the test stimuli; (iii) better fits were found for responses in the face recognition task in North American students, while a better fit for responses in the name recognition task was found in Spanish students.

These results are of interest, given the relationship found between face and word recognition processes. In this way, not only face recognition might occur faster, but an interaction between face and word name recognition has also been found, indicating diverse patterns of recognition. These results might shed light on the relationship found between face and word recognition processes. It should be noted that studies with developing readers have shown a decrease in face processing in favor of written letter feature recognition [49]. This result is also congruent with previous literature in clinical groups, such as participants with dyslexia [50] or autism spectrum disorder [51], where one process is impaired while the other one seems to be preserved. A very common interpretation justifies that the process of word recognition, which is a learned process, takes place in an area of the brain not intended for this purpose. In this case, word recognition development would be detrimental for face processing. This last one is considered innate and developed in the same brain area. However, recent work suggests that specialization for each process, which rather than being independent, might be bilaterally distributed with some preferences [48,52]. This explanation is supported by the development of specialized area for each process, as described in the introduction of this work. Moreover, this explanation also makes the differences between the two countries under study, and ultimately, the effects described in the literature on the hometown of origin, plausible [29,30]. Depending on the type and amount of stimulus that one comes across in one’s daily routine, the specialization of the underlying areas for each process would be developed.

Another possible explanation for the differences found could lie with the approaches that relate specific attentional and executive mechanisms. As previously mentioned, some positions suggest that face processing is automatic [19,20,21,22,23], based on the familiarity variable or holistic perspective for face processing, while other approaches suggest the existence of separate attentional resources inherent to perceptual resources. In contrast, the literature seems to support the attentional independence of word recognition, as the VWFA has been described for face recognition as part of the language and attention circuitry [25]. Recent literature casts doubt on the holistic perspective and argues that the exponential component (*τ*), obtained through an ex-Gaussian analysis, is related to working memory and attentional processes [53,54]. However, note that in our case, no remarkable differences were found in the τ parameter between the different conditions under study. In other words, differences in the parameter were not bigger than the sum of the associated uncertainties (*σ*). Nevertheless, the particular interpretation of the ex-Gaussian components as a cognitive reflex is a very controversial one in the literature, and caution is advised here [55]. It should be noted that these parameters are not isolated, but interrelated [45]. One of the main limitations of this study is not measuring the attentional levels of the participants from different assessment techniques, and its relationship to ex-Gaussian components, an aspect of interest for future research within this field.

Through the present methodology, the role of processing components might shed light, as network analyses have done previously in the field [34,56]. It must be considered here that response latencies generally show a high sensitivity to cognitive processes, but their distribution is often positively distributed. This is not only problematic for some statistical analysis methods, but also, in terms of signal detection theories, certain scores can be confused with noise, just as noise can be confused with valid scores, also called signal [57]. For all these reasons, and unlike other studies, the ex-Gaussian technique allowed us to analyze response times without applying any trimming technique or transformation because of the skewness distribution of these data. Furthermore, the literature seems to support that behavioral response latencies adequately fit an ex-Gaussian distribution [58]. Even if the ex-Gaussian fit is not a new technique, an innovative aspect is its application is proposed in this paper. While the scientific literature is extensive in the use of ex-Gaussian fits for processes related to response analysis in word recognition tasks, the number of papers for face recognition processes using the ex-Gaussian fit is smaller. By this, we do not mean that this analysis is specific for word or pattern recognition processes, but of interest for other underexplored areas in the area. Our results show that its fit is improved according to participant’s profile. In other words, there are underlying variables such as culture, or perhaps mother language, which may interact in the recognition of faces and words. Future lines of research should systematically address whether it is possible that these variables interfere in the quality of the fit ex-Gaussian fit.

Another limitation of the study is that not all the perceptual characteristics of the stimuli have been explored. Faces have been presented in black and white resolution to facilitate comparisons with names, but in their more ecological environment, faces vary in color, expression, and position [59,60]. Ultimately, this question could be related to theories of interest to entropy approaches. On the other hand, words vary in lexical aspects, which have been tried to be controlled through what is considered a pre-lexical task, in this case, a masked priming task. Further lines of research should address the role of these variables in human recognition.

Lastly, we consider that these results could be of interest at both theoretical and applied levels. First, let us recall that the most relevant models in the field have identified different processing stages [26], for the identity of a stimulus, where the interaction between processes and their familiarity could be included (in our results for both test and distractor stimuli). At the applied level, information in this area is of interest for intervention programs for deficits associated with face and word recognition.

## Figures and Tables

**Table 1:** Descriptive analysis and ex-Gaussian components across conditions and countries in face and word recognition.

			Prime	*Hit (%)*	*M*	*S*	*t*	*µ*	*σ*	*τ*	*p*
Target Face	Spain	Test	Identity	81	645.57	228.38	1.98	417.56	18.72	227.72	0.00
			Related	83	676.03	229.20	1.99	446.41	17.37	228.81	0.00
			Unrelated (same nature)	83	697.92	225.71	1.86	498.62	77.04	199.30	0.00
			Unrelated (different nature)	78	691.99	222.01	1.61	481.05	61.48	210.93	0.01
		Distracting	Identity	80	706.13	255.76	1.70	468.39	70.74	237.74	0.04
			Related	87	728.19	242.00	1.72	502.11	58.64	226.07	0.00
			Unrelated (same nature)	86	716.47	255.16	1.61	483.74	84.50	232.73	0.00
			Unrelated (different nature)	86	737.74	238.61	1.77	520.84	71.68	216.90	0.00
	USA	Test	Identity	71	671.15	222.76	1.66	456.03	56.71	215.11	0.79
			Related	76	699.16	252.74	2.01	451.39	36.49	250.15	0.00
			Unrelated (same nature)	73	725.25	227.87	1.85	509.77	54.78	215.48	0.08
			Unrelated (different nature)	69	728.80	246.94	1.48	482.41	44.27	246.38	0.52
		Distracting	Identity	80	713.38	267.79	1.58	463.75	80.76	249.63	0.01
			Related	87	723.93	252.98	1.75	498.38	84.62	225.55	0.00
			Unrelated (same nature)	83	701.19	259.20	1.68	469.98	87.87	231.21	0.00
			Unrelated (different nature)	86	732.96	243.38	1.58	509.47	82.87	223.49	0.03
Target Name	Spain	Test	Identity	79	723.52	252.40	1.77	483.75	67.10	239.76	0.06
			Related	80	733.43	245.07	1.60	498.17	63.08	235.26	0.29
			Unrelated (same nature)	80	776.03	242.95	1.59	565.34	96.59	210.69	0.00
			Unrelated (different nature)	80	780.31	261.58	1.57	532.32	80.38	247.98	0.68
		Distracting	Identity	86	866.06	298.14	1.10	582.47	119.21	283.59	0.14
			Related	85	903.43	286.98	1.21	615.45	93.19	287.98	0.20
			Unrelated (same nature)	87	924.25	300.37	1.03	614.45	95.48	309.80	0.05
			Unrelated (different nature)	86	870.07	299.21	1.19	563.35	87.99	306.72	0.03
	USA	Test	Identity	78	700.25	257.88	1.94	468.30	60.72	231.95	0.00
			Related	77	718.87	258.20	1.85	489.53	72.59	229.43	0.00
			Unrelated (same nature)	78	762.75	246.69	1.96	537.22	57.63	225.53	0.01
			Unrelated (different nature)	78	745.48	268.94	1.92	506.51	67.87	238.97	0.00
		Distracting	Identity	90	794.54	301.97	1.39	502.44	87.16	292.10	0.04
			Related	91	823.69	287.05	1.55	555.94	88.33	267.74	0.02
			Unrelated (same nature)	91	822.75	285.69	1.41	547.36	86.83	275.37	0.02
			Unrelated (different nature)	88	784.81	277.96	1.38	517.38	88.40	267.42	0.09

## Data Availability

Not Applicable.

## Appendix A

Test stimuli employed from previous research [34]. Google frequency search was obtained following the same procedure than previous literature [40,43].

**Celebrity**	**Google Frequency Search**
Adele	139,000,000
Amy Winehouse	25,200,000
Angelina Jolie	230,000,000
Bill Clinton	187,000,000
Brad Pitt	136,000,000
Britney Spears	161,000,000
Beyoncé	207,000,000
Eddie Murphy	83,000,000
Elvis Presley	125,000,000
Freddie Mercury	68,300,000
George Clooney	29,600,000
Jack Nicholson	76,800,000
Jessica Alba	96,300,000
Keanu Reeves	34,600,000
Kevin Bacon	44,400,000
Leonardo DiCaprio	52,400,000
Madonna	199,000,000
Marilyn Monroe	143,000,000
Morgan Freeman	113,000,000
Naomi Campbell	77,900,000
Prince Harry	429,000,000
Rihanna	189,000,000
Salma Hayek	37,200,000
Scarlett Johansson	112,000,000
Shakira	114,000,000
Sigourney Weaver	8,520,000
Silvio Berlusconi	15,000,000
Winona Ryder	29,900,000
